# Knockdown of Mtfp1 can minimize doxorubicin cardiotoxicity by inhibiting Dnm1l‐mediated mitochondrial fission

**DOI:** 10.1111/jcmm.13250

**Published:** 2017-06-23

**Authors:** Lynn H. H. Aung, Ruibei Li, Bellur S. Prabhakar, Peifeng Li

**Affiliations:** ^1^ Department of Microbiology and Immunology College of Medicine University of Illinois at Chicago Chicago IL USA; ^2^ School of Professional Studies Northwestern University Chicago IL USA

**Keywords:** mitochondrial fission process 1 (Mtfp1), dyanmic‐1‐like (Dnm1l), mitochondrial fission, doxorubicin, cardiotoxicity

## Abstract

The long‐term usage of doxorubicin (DOX) is largely limited due to the development of severe cardiomyopathy. Many studies indicate that DOX‐induced cardiac injury is related to reactive oxygen species generation and ultimate activation of apoptosis. The role of novel mitochondrial fission protein 1 (Mtfp1) in DOX‐induced cardiotoxicity remains elusive. Here, we report the pro‐mitochondrial fission and pro‐apoptotic roles of Mtfp1 in DOX‐induced cardiotoxicity. DOX up‐regulates the Mtfp1 expression in HL‐1 cardiac myocytes. Knockdown of Mtfp1 prevents cardiac myocyte from undergoing mitochondrial fission, and subsequently reduces the DOX‐induced apoptosis by preventing dynamin 1‐like (Dnm1l) accumulation in mitochondria. In contrast, when Mtfp1 is overexpressed, a suboptimal dose of DOX can induce a significant percentage of cells to undergo mitochondrial fission and apoptosis. These data suggest that knocking down of Mtfp1 can minimize the cardiomyocytes loss in DOX‐induced cardiotoxicity. Thus, the regulation of Mtfp1 expression could be a novel therapeutic approach in chemotherapy‐induced cardiotoxicity.

## Introduction

Doxorubicin (DOX), an anthracycline chemotherapeutic agent, has been widely used in various cancer treatment regimens. However, its potential usage in long term is largely limited due to the development of severe cardiomyopathy, and in some cases, was reported to be fatal 1, 2, 3. Although multiple mechanisms interplay in DOX‐induced cardiomyopathy, many studies consistently indicate that DOX‐induced cardiac injury is related to reactive oxygen species generation (ROS). DOX exposure increases free radical formation, and at the same time, causes a decrease in endogenous antioxidant response, which results in an increase in oxidative stress 4, 5, 6. The increase in cell stress mediated by ROS leads to an alteration in mitochondrial dynamics and activates apoptotic signalling 7, 8, 9.

Being terminally differentiated cells, cardiomyocytes possess a limited regeneration capacity. Apoptosis plays a crucial role in cardiac physiology and pathology. For instance, during embryonic development, apoptosis is essential for the morphogenesis and developmental remodeling of cardiac tissues while in later life, it is a prerequisite for removing damaged or impaired cardiomyocytes to maintain normal cardiac function 10, 11. Likewise, apoptosis is associated with major cardiomyocytes loss in several cardiac diseases including myocardial ischaemia and infarction, cardiac hypertrophy, heart failure and DOX‐induced cardiotoxicity 11, 12, 13. Thus, it is critical to understand the molecular mechanism underlying the DOX‐induced cardiomyocyte apoptosis to effectively prevent cardiomyocyte loss during DOX treatment.

Cardiomyocytes are highly energy consuming cells; therefore, they are enriched with mitochondria 14, 15. Recently, it has been demonstrated that mitochondrial morphology determines the mitochondrial function 16, 17, 18. In several cardiac diseases, the mitochondrial morphology in cardiac myocytes was found to be distorted 19. Fragmentation of mitochondria increases the mitochondrial membrane permeability and initiates the release of mitochondrial pro‐apoptotic factors such as cytochrome‐c (Cyt‐c), which further activates the downstream apoptotic cascades 20, 21. Although several studies have reported an association of mitochondrial fission with cardiac apoptosis, the exact underlying molecular mechanisms are still not fully understood.

Mitochondrial membrane proteins such as DRP1 and hFis1 are the key mediators of mitochondrial fission 22, 23. However, the molecular mechanism of their actions has not been fully understood. Recently, a novel mitochondrial inner membrane protein, mitochondrial fission protein 1 (Mtfp1), also known as Mtp18, has been identified 24 and was shown to be essential for maintenance of mitochondrial integrity and thus has been implicated in the regulation of mitochondrial fission in cancer cells 25. However, it remains unknown as to whether Mtfp1 plays a role in cardiomyocyte mitochondrial fission process. More importantly, no previous study has tested whether Mtfp1 participates in the process of DOX‐induced mitochondrial fission and apoptosis.

Therefore, our current study was aimed at uncovering the role Mtfp1 in the regulation of DOX‐induced mitochondrial fission and cardiac myocyte apoptosis using HL‐1 cell line. Our work shows that Mtfp1 might play a pro‐apoptotic role, and knockdown of Mtfp1 can prevent cardiomyocyte loss in DOX‐induced cardiotoxicity by affecting Dnm1l‐mediated mitochondrial fission. Taken together, this study provides an additional evidence to better understand the therapeutic potential of mitochondrial protein in chemotherapy‐induced cardiotoxicity.

## Materials and methods

### Cell cultures and doxorubicin treatment

HL‐1 cardiac cell line was kindly provided by Dr. Xing Rong (Institute of Cardiovascular Development and Translational Medicine, Wenzhou Medical University, Zhejiang, China). HL‐1 is a cardiac muscle cell line, derived from the AT‐1 mouse atrial cardiomyocyte tumour lineage. This cell line has been shown to retain the phenotypic characteristics of cardiomyocytes even after serial passage 26. They were cultured in Claycomb media supplemented with 10% foetal bovine serum (Sigma‐Aldrich, St. Louis, MO, USA), 0.1 mol/l noradrenaline (Sigma‐Aldrich), 2 mmol/l L‐glutamine (Invitrogen, Carlsbad, CA, USA) and penicillin/streptomycin (Invitrogen) in a humidified 5% CO2 incubator at 37°C 26. Doxorubicin was purchased from Sigma‐Aldrich. The treatment with doxorubicin was performed as we have described earlier 27.

### Lentiviral construct of Mtfp1‐shRNA and infection

Mtfp1‐shRNA and control shRNA lentivirus based plasmid were purchased from Origene (Rockville, MD, USA). Viruses were amplified in 293T cells. Cells were infected with the virus according to the manufacturer's protocol. Mtfp1‐shRNA contains four specific constructs, encoding shRNA designed to knock down Mtfp1 expression, and control shRNA lentiviral‐based plasmid contains an shRNA construct encoding a scrambled sequence that will not lead to specific degradation of any known cellular mRNA. The Mtfp1 shRNA: the Mtfp1‐shRNA‐A sequence was 5′‐CTTTGTATGGCAGGCTCTAGCCTCTGTGG‐3′, Mtfp1‐shRNA‐B sequence was 5′‐CCATTGACAGGTCGGTAGACTTCCTCCTG‐3′, Mtfp1‐shRNA‐C sequence was 5′‐TCCAGCTCCTATGTCTTGGCCGATGCCAT‐3′, and Mftp1‐shRNA‐D sequence was 5′‐AGAAGGCAGGAGAGGTGCCAAGCCCTGAA‐3′. Among four different shRNA plasmids, Mtfp1‐shRNA‐D showed most significant knockdown effect on Mtfp1 expression. Therefore, we chose to use Mtfp1‐shRNA‐D in the subsequent experiments. The scrambled shRNA sense sequence was 5′‐GCACTACCAGAGCTAACTCAGATAGTACT‐3′, and the antisense sequence was 5′‐AGTACTATCTGAGTTAGCTCTGGTAGTGC‐3′. Cells were infected with the virus according to the manufacturer's protocol. The Mtfp1 expression levels were checked by Western blot.

### Lenti ORF clone of Mtfp1 construct and infection

Lentiviral particle harbouring the cDNA of Mtfp1 (RR203770L2) was purchased from Origene. Viruses were amplified in 293T cells. Cells were infected with the virus according to the manufacturer's protocol. The Mtfp1 expression levels were checked by Western blot.

### Dnm1l siRNA transfection

The Dnm1l siRNA and control siRNA‐A (scrambled siRNA) were purchased from Santa Cruz Biotechnology, Inc. (Dallas, TX, USA). Dnm1l siRNA is a pool of three target‐specific 19‐ to 25‐nt siRNAs designed to knock down Dnm1l gene expression. For Dnm1l siRNA‐A, the sense sequence is 5′‐CAGUAUCAGUCUCUUCUAAtt‐3′ and antisense is 5′‐UUAGAAGAGACUGAUACUGtt‐3′; for Dnm1l siRNA‐B, the sense sequence is 5′‐CAUCUUGACCGCCAUUAGAtt‐3′ and antisense is 5′‐CAUCUUGACCGCCAUUAGAtt‐3′; for Dnm1l siRNA‐C, the sense sequence is 5′‐GUAUCGCGAGACAAGUUAAtt‐3′ and antisense is 5′‐UUAACUUGUCUCGCGAUACtt‐3′. For scrambled siRNA‐A, the sense sequence is 5′‐CAGUAUCAGUCUCUUCUAAtt‐3′ and antisense is 5′‐ACGUGACACGUUCGGAGAAtt‐3′. Cells were seeded 24 hrs before transfection and were then transfected with 60 nM scrambled siRNA or Dnm1l siRNA using Lipofectamine^®^ 2000 Reagent (Invitrogen) as per the manufacturer's instructions.

### Immunoblotting

Immunoblotting was performed as we have reported earlier 28. In brief, cells were lysed for 1 hr at 4°C in RIPA buffer [20 mmol/l Tris (pH 7.5), 2 mmol/l EDTA, 3 mmol/l EGTA, 2 mmol/l DTT, 250 mmol/l sucrose, 0.1 mmol/l phenylmethylsulfonyl fluoride (PMSF), 1% Triton X‐100 and a protease inhibitor cocktail]. The samples were separated by sodium dodecyl sulphate–PAGE (SDS–PAGE) using a 4–20% Mini‐PROTEAN TGX™ Precast Gel (Bio‐Rad, Hercules, CA, USA) and transferred for 1 hr at 80 V to PVDF membrane (Merck Millipore Ltd, Darmstadt, Germany). Equal‐protein loading was controlled by Ponceau red staining of membranes. The membrane was blocked in Tris‐buffered saline containing 5% milk and 0.1% of Tween‐20 for 1 hr, and then incubated overnight with primary antibody. Blots were probed with horseradish peroxidase‐conjugated goat anti‐rabbit IgG or goat anti‐mouse IgG. (Santa Cruz Biotechnology, Inc., Dallas, TX, USA). Antigen–antibody complexes were visualized by Amersham™ ECL™ Prime Western Blotting Detection Reagent (GE Healthcare, Buckinghamshire, UK). Anti‐MTFP1, anti‐caspase‐3 polyclonal and anti‐cleaved PARP monoclonal antibodies were from Abcam (Cambridge, MA, USA). Anti‐tubulin polyclonal, and anti‐cyclooxygenase IV and anti‐cytochrome‐c monoclonal antibodies were from Cell Signaling Technology, Inc. (Danvers, MA, USA). Anti‐actin monoclonal and anti‐DNM1L polyclonal antibodies were from Santa Cruz Biotechnology, Inc. The protein band intensity was quantified by ImageJ (National Institutes of Health, Bethesda, MD, USA) using protocol written by Luke Miller, November 2010 (http://www.lukemiller.org/ImageJ_gel_analysis.pdf). Briefly, the density of each sample was first quantified with image J, and then the per cent value of each sample and that of standard was calculated. Finally, the relative density was calculated by dividing the per cent value of each sample by the per cent value of each standard 28.

### Immunoprecipitation

Immunoprecipitation was carried out as described earlier 29. In brief, cells were lysed for 1 hr at 4°C in a lysis buffer. The cell lysates were precleared with 10% (vol/vol) protein A‐agarose (Roche, Branford, CT, USA) for 1 hr on a rocking platform. Specific antibodies were added and rocked for overnight. Immunoprecipitates were captured with 10% (vol/vol) protein A‐agarose for another hour. The agarose beads were spun down and washed thrice with NET buffer. The antigens were released and denatured by adding SDS sample buffer.

### DNA fragmentation and apoptosis assays

DNA fragmentation was monitored using the cell death detection ELISA kit (Roche) as we have described elsewhere 29. Briefly, the anti‐histone monoclonal antibody was added to the 96‐well ELISA plates and incubated overnight at 4°C. After recoating and rinsing three times, the cytoplasmic fractions were added and incubated for 90 min. at room temperature. After three washes, bound nucleosomes were detected by the addition of anti‐DNA peroxidase monoclonal antibody and reacted for 90 min. at room temperature. After the addition of the substrate, the optical density was determined at 405 nm using an ELISA plate reader. For apoptosis analysis, a terminal deoxynucleotidyl transferase‐mediated Dutp nick‐end‐labelling (TUNEL) kit from Abcam (Cambridge, MA, USA) was used. After transfection and treatment as indicated, cells were rinsed, fixed, permeabilized and stained with the *in situ* BrdU‐red DNA fragmentation TUNEL assay according to the kit's instructions. Images were taken using a laser scanning confocal microscope (Zeiss LSM 710 BIG, Dublin, CA, USA). Two hundred and fifty to three hundred cells were counted in 20–30 random fields in each group. Results are expressed as percentage of TUNEL‐positive cells.

### Preparation of mitochondrial fractions

Mitochondrial fractions were prepared as we have described earlier 29. Briefly, cells were washed twice with PBS and the pellet was suspended in 0.2 ml of buffer A (20 mM HEPES pH 7.5, 10 mM KCl, 1.5 mM MgCl_2_, 1 mM EGTA, 1 mM EDTA, 1 mM DTT, 0.1 mM PMSF, 250 mM sucrose) containing a protease inhibitor cocktail (Sigma‐Aldrich). The cells were homogenized by 12 strokes in a Dounce homogenizer. The homogenates were centrifuged twice at 750 *g* for 5 min. at 4°C to collect nuclei and debris. The supernatants were centrifuged at 10,000 *g* for 15 min. at 4°C to collect mitochondria‐enriched heavy membranes (HM). The resulting supernatants were centrifuged to yield cytosolic fractions.

### Analysis of mitochondrial fission

Mitochondrial fission was analysed by staining mitochondria as we and others have described earlier with some modification 30. Briefly, cells were plated onto the coverslips. After treatment, they were stained for 15 min. with 100 nM MitoTracker Red CMXRos (Molecular Probes, Eugene, OR, USA). Cells were fixed in 4% paraformaldehyde for 15 min. and permeabilized with 0.2% Triton X‐100. Mitochondria were imaged using a laser scanning confocal microscope (Zeiss LSM 710 BIG, Dublin, USA). The detailed procedure of analysis of mitochondrial morphology was as described 30. Cells with disintegrated mitochondria were taken as mitochondrial fission. The percentage of cells with fragmented mitochondria relative to the total number of cells is presented as the mean ± SEM of at least three independent experiments, counted by an observer blinded to the experimental conditions; 200–300 cells in 20–30 random fields per group were counted.

### Prediction of a potential Mtfp1's target protein

The potential target protein was predicted using STRING v10 (http://string-db.org/cgi/input.pl). The search term was set as ‘Mtfp1’ and organism as ‘Mus musculus’. The protein–protein interaction was determined by the interaction score, which is an indicator of confidence regarding how likely STRING judges an interaction to be true, given the available evidence. The score can range from 0 to 1, with 1 being the highest possible confidence 31.

### Statistical analysis

Data are expressed as the mean ± SEM of at least three independent experiments for each experimental group. We evaluated the data with Student's *t*‐test for comparisons between two groups and a one‐way analysis of variance (ANOVA) for multiple comparisons. Statistical analyses were performed with GraphPad Prism 5.0 (GraphPad Software, Inc., San Diego, CA, USA). A value of *P *< 0.05 was considered significant.

## Results

### DOX induces apoptosis in HL‐1 cardiac myocytes

**Figure 1:**
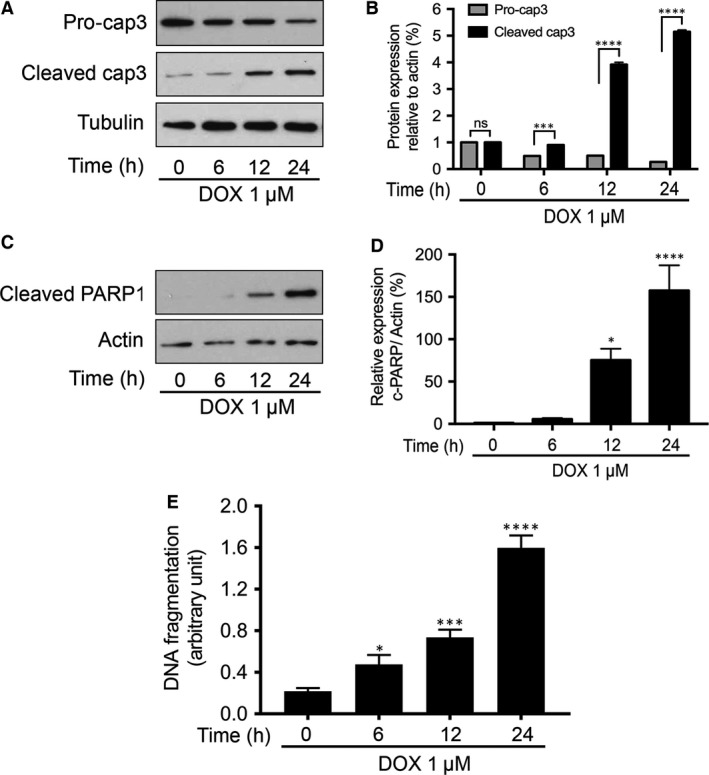
Doxorubicin induces apoptosis in HL‐1 cardiac myocytes. **A** and **B** show caspase‐3 cleavage by immunoblot and densitometry; **C** and **D** show PARP1 cleavage by immunoblot and densitometry. Tubulin and β‐actin served as a loading control. (**A**–**D**) Cells were stimulated with 1 μmol/l DOX and then harvested at the indicated time for immunoblotting. Figures presented are the representative figures of at least three independent experiments. The densitometry data were expressed as the mean ± SEM of three independent experiments. **E** shows DNA fragmentation. Cells were stimulated with 1 μmol/l DOX at indicated time‐points and apoptosis‐related DNA fragmentation were analysed using the cell death detection ELISA (**E**). Data were expressed as the mean ± SEM of three independent experiments. ns, non‐significant, *P < 0.05, ***P < 0.001, ****P < 0.0001 *versus* 0 hr.

Mtfp1 has been shown to play an important role in the regulation of mitochondrial fission in various cancer cells 24, 25. However, its role in cardiomyocytes remains largely unknown. To understand whether Mtfp1 is involved in the regulation of DOX‐induced cardiomyocyte mitochondrial fission and apoptosis, we treated the HL‐1 cells with DOX (1 μmol/l) and determined the extent of apoptosis. We detected the level of cleavage of pro‐caspase 3 and poly‐ADP ribose polymerase (PARP) at different durations of DOX treatment. We observed a time‐dependent increase in the cleavage of caspase‐3 (Fig. 1A and B) and PARP upon DOX exposure (Fig. 1C and D). A time‐dependent increase in apoptosis‐related DNA fragmentation was also observed (Fig. 1E).

### Doxorubicin‐induced mitochondrial fission is associated with the up‐regulation in Mtfp1 expression

**Figure 2:**
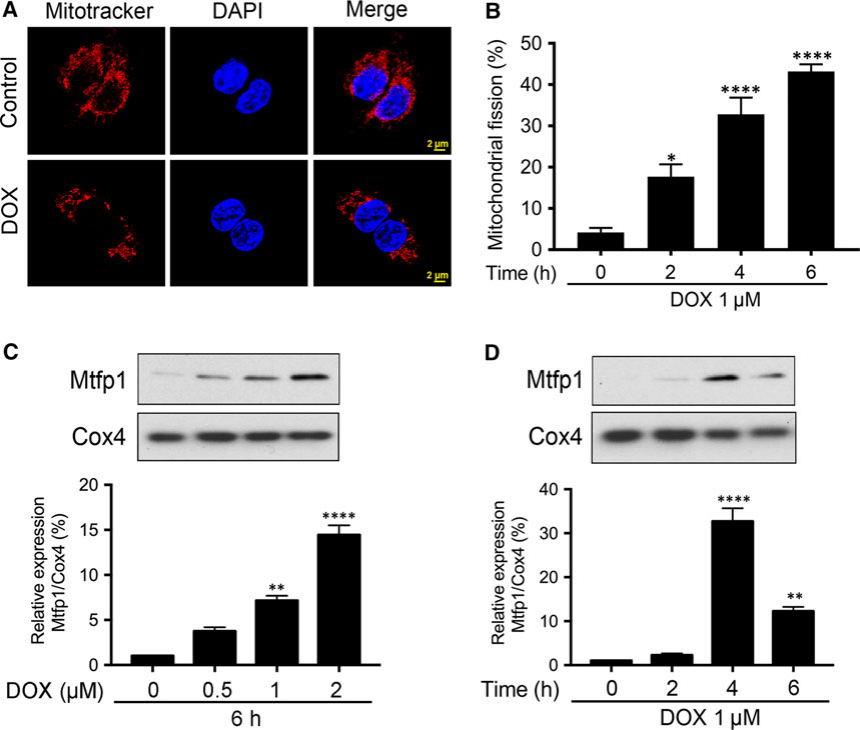
Doxorubicin‐induced mitochondrial fission is associated with up‐regulation in Mtfp1 expression. (**A** and **B**) doxorubicin (DOX) induces mitochondrial fission in HL‐1 cells. Cells were stimulated with 1 μmol/l DOX at indicated time‐points and mitochondrial morphology was analysed. **A** shows mitochondrial morphology. **B** shows percentage of cells undergoing mitochondrial fission. Data were expressed as the mean ± SEM of three independent experiments. (**C** and **D**) DOX up‐regulates mitochondrial fission process 1 (Mtfp1) expression in mitochondria in a dose‐ and time‐dependent manner. Analysis of Mtfp1 expression. HL‐1 cells were stimulated with the indicated doses of DOX and harvested at 6 hrs (**C**,* upper panel*), and cells were stimulated with 1 μmol/l DOX and then harvested at the indicated time (**D**,* upper panel*) for immunoblotting. Cytochrome‐c oxidase (Cox4) served as a loading control for mitochondrial fraction. Figures presented are the representative figures of at least three independent experiments. The densitometry data were expressed as the mean ± SEM of three independent experiments (**C** and **D** *lower panels*). *P < 0.05, **P < 0.01, ****P < 0.0001 *versus* non‐treatment.

As shown in Figure 2A, compared to negative control (where the mitochondria are long, thin, filamentous), the DOX‐treated group displayed punctate disintegrated mitochondria, which is regarded as fission. In quantitative analysis, a time‐dependent increase in the percentages of cells with mitochondrial fission upon DOX exposure was observed (Fig. 2B). These findings confirmed that DOX induces mitochondrial fission and apoptosis in HL‐1 cells. At the same time, we observed an up‐regulation of Mtfp1 expression upon DOX exposure (Fig. S1). Then, we tested the mitochondrial expression of Mtfp1 by preparing subcellular fractions. Our results showed that DOX up‐regulated Mtfp1 expression in mitochondria in a time‐ and dose‐dependent manner (Fig. 2C and D), suggesting that Mtfp1 may be involved in the regulation of DOX‐induced mitochondrial fission and apoptosis in HL‐1 cells.

### Knockdown of Mtfp1 can prevent the induction of mitochondrial fission upon DOX exposure and can inhibit apoptosis

**Figure 3:**
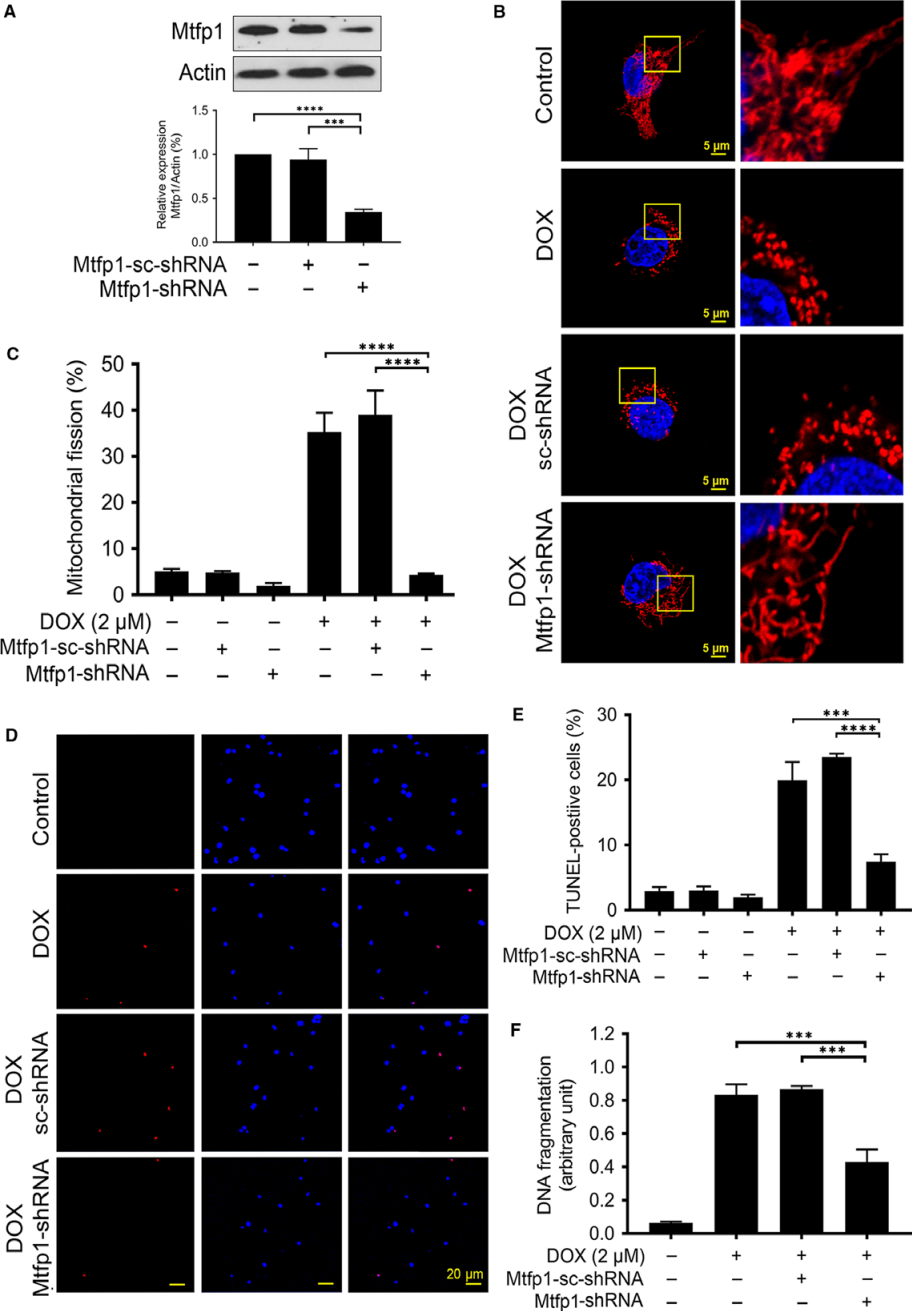
Knockdown of Mtfp1 can prevent HL‐1 cell from doxorubicin‐induced mitochondrial fission and apoptosis. (**A**) Analysis of mitochondrial fission process 1 (Mtfp1) expression. Immunoblot shows Mtfp1 knockdown in HL‐1 cells. β‐actin served as a loading control. The densitometry data were expressed as the mean ± SEM of three independent experiments (*lower panel*). **B** shows mitochondrial morphology. Bar = 2 μm. **C** shows percentage of cells with mitochondrial fission. Cells were exposed to a higher concentration (2 μmol/l) doxorubicin (DOX) and mitochondrial fission was analysed by MitoTraker Red (**B** and **C**). **D** shows TUNEL‐positive cells. Bar = 20 μm. **E** shows percentage of TUNEL‐positive cells. **F** shows apoptosis‐related DNA fragmentation. Cells were exposed to a higher concentration (2 μmol/l) DOX, and apoptosis was analysed by TUNEL assay (**D** and **E**), and DNA fragments were analysed using the cell death detection ELISA (**F**). Figures presented are the representative figures of at least three independent experiments. Data were expressed as the mean ± SEM of three independent experiments. ***P < 0.001 and ****P < 0.0001.

To understand the importance of Mtfp1 in DOX‐induced cardiotoxicity, we first knocked down the endogenous Mtfp1 expression using Mtfp1‐shRNA. The expression level of Mtfp1 was significantly reduced by its shRNA but not its scrambled form (Fig. 3A). We then tested whether Mtfp1 is related to the occurrence mitochondrial fission. In knockdown group, the mitochondria appear in long thin filamentous form, as compared to punctate structure in cells that were left untransfected or transfected with inactive scrambled shRNA. Knockdown of Mtfp1 significantly reduced percentage of cells undergoing mitochondrial fission compared to its negative or scrambled controls even with high concentration (2 μmol/l) of DOX exposure (Fig. 3B and C).

To further understand whether changes in mitochondrial dynamic due to Mtfp1 modulation can influence apoptosis, we detected the extent of apoptosis in HL‐1 cells with reduced Mtfp1 expression upon exposure to high dose of DOX. We observed a significant reduction in DOX‐induced apoptosis in Mtfp1 knockdown group, compared to its scrambled control, as observed by TUNEL assay (Fig. 3D and E). We further confirmed the pro‐apoptotic effect of Mtp1 by employing cell death ELISA and found that there was a consistent reduction in DNA fragmentation in Mtfp1 knockdown group relative to its positive and negative controls upon high‐dose DOX exposure (Fig. 3F). These data suggested that Mtfp1 plays a pro‐mitochondrial fission and pro‐apoptotic role in DOX‐induced cardiotoxicity.

### Overexpression of Mtfp1 promotes HL‐1 cells to undergo doxorubicin‐induced mitochondrial fission and apoptosis

**Figure 4:**
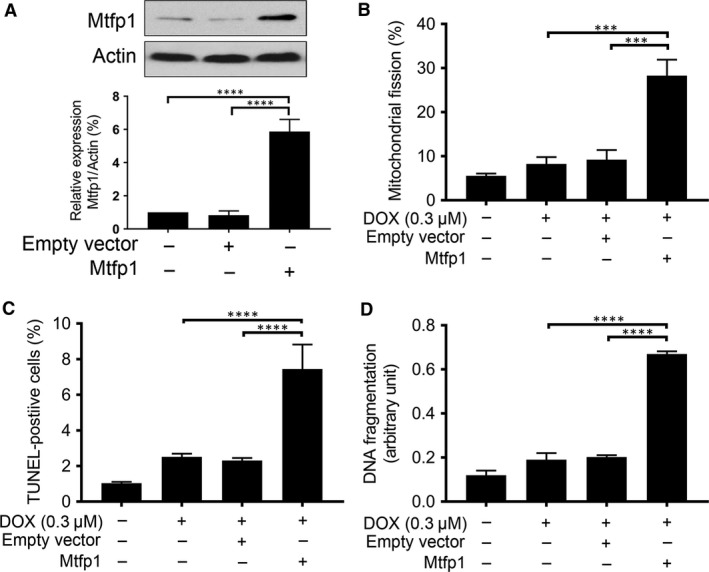
Enforced expression of Mtfp1 sensitizes HL‐1 cells to doxorubicin‐induced mitochondrial fission and apoptosis. (**A**) Analysis of Mtfp1 expression. Immunoblot shows Mtfp1 overexpression in HL‐1 cells (*upper panel*). β‐actin served as a loading control. The densitometry data were expressed as the mean ± SEM of three independent experiments (*lower panel*). (**B**) Enforced expression of Mtfp1 sensitizes cells to undergo DOX‐induced mitochondrial fission. HL‐1 cells were exposed to a lower concentration of DOX. **B** shows percentage of cells with mitochondrial fission; (**C** and **D**) Enforced expression of Mtfp1 sensitizes cells to undergo DOX‐induced apoptosis. Cells were exposed to a lower concentration (0.3 μmol/l), and percentages of apoptosis were analysed by TUNEL assay (**C**) and DNA fragments were analysed using the cell death detection ELISA (**D**). Data were expressed as the mean ± SEM of three independent experiments. Figures presented are the representative figures of at least three independent experiments. ***P < 0.001 and ****P < 0.0001.

We hypothesize that if Mtfp1 is pro‐mitochondrial fission and pro‐apoptotic, then subcytotoxic dose of DOX should be able to induce mitochondrial fission and apoptosis when Mtfp1 is overexpressed. To test this hypothesis, we infected the cells with lentiviral construct of Mtfp1‐cDNA to induce Mtfp1 expression (Fig. 4A). After 24 hrs, the cells were treated with subcytotoxic dose of DOX for 6 hrs and then the percentages of cells with mitochondrial fission and apoptosis were analysed. We found that a low concentration DOX (0.3 μmol/l) can induce a significantly higher number of cells to undergo mitochondrial fission in Mtfp1 overexpressed group; on the other hand, it did not cause any significant change in the percentage of mitochondrial fission in the negative and empty vector control groups (Fig. 4B). Concomitantly, the suboptimal concentration of DOX could induce a significantly higher percentage of apoptosis in Mtfp1 over‐expressed group relative to its negative and empty vector controls (Fig. 4C and D).

### Dynamin 1‐like protein (Dnm1l) is predicted to be a Mtfp1's target protein

**Figure 5:**
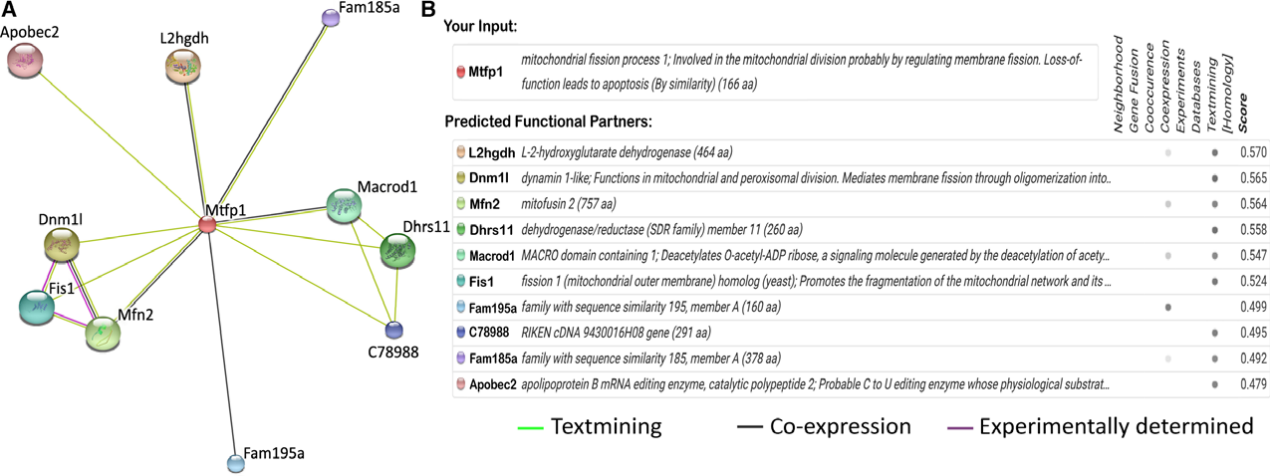
Dynamin 1‐like protein (Dnm1l) is predicted to be a Mtfp1's target protein. **A** shows schematic representation of the Mtfp1's target protein interaction network. **B** shows the confidence interaction scores of potential functionally associated proteins. Protein interaction network was constructed by STRING v10. Dnm1l shows highest estimated confidence score among all the potential functionally associated proteins.

To understand the molecular mechanism, we performed protein interaction analysis to predict the target protein exhibiting the highest possible functional correlation with Mtfp1 32. As in Figure 5A and B, the string search output revealed a total of 10 proteins which showed a medium or high confidence score of interaction (≥0.400). Of which, L‐2‐hydroxyde hydroxyglutarate dehydrogenase (L2hgdh) scored the highest followed by dynamin 1‐like (Dnm1l) and Mitofusin2 (Mfn2). As this study was focused on molecular mechanism of mitochondrial fission, we paid closer attention to a protein that has strong correlation with mitochondrial fission 27, 33, 34. Therefore, we selected Dnm1l for further investigation.

### Dnm1l is required for doxorubicin to induce mitochondrial fission and Mtfp1 promotes doxorubicin‐induced Dnm1l accumulation in mitochondria

**Figure 6:**
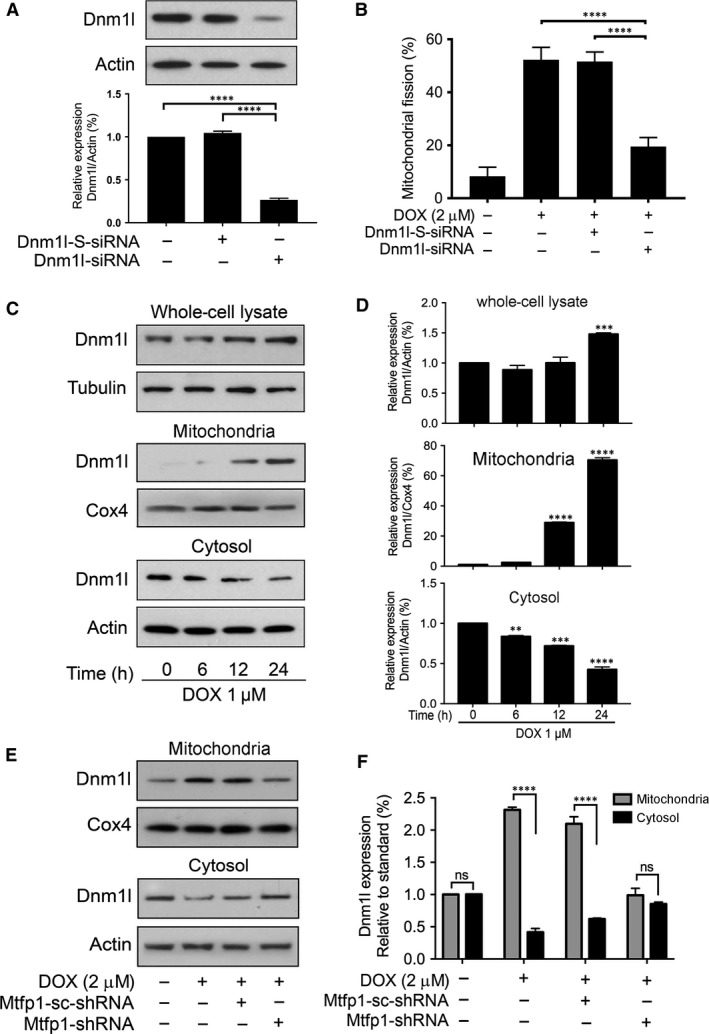
Dnm1l is required for doxorubicin‐induced mitochondrial fission and Mtfp1 promotes doxorubicin‐induced Dnm1l accumulation in mitochondria. (**A** and **B**) Dnm1l is required for DOX to induce mitochondrial fission. (**A**) Analysis of Dnm1l expression. Immunoblot shows Dnm1l knockdown in HL‐1 cells. HL‐1 cells were transfected with scrambled siRNA, or Dnm1l siRNA, respectively, and after 48 hrs, were harvested for immunoblot (*upper panel*). Lower panel shows the densitometry. β‐actin served as a loading control. (**B**) Knockdown of Dnm1l inhibits doxorubicin (DOX)‐induced mitochondrial fission. **B** shows percentage of cells with mitochondrial fission. (**C** and **D**) DOX induces translocation of Dnm1l from cytosol to mitochondria. **C** shows Dnm1l expression by immunoblot. **D** shows the densitometry. **P < 0.01, ***P < 0.001 and ****P < 0.0001 *versus* 0 hr. (**E** and **F**) Knockdown of Mtfp1 inhibits DOX‐induced Dnm1l accumulation in mitochondria. Then, they were treated with a higher concentration (2 μmol/l) of DOX for 24 hrs and harvested for subcellular fraction, and protein expression levels in different cellular compartments were analysed by immunoblot. **E** shows Dnm1l expression in subcellular fraction and **F** shows densitometry. The densitometry data were expressed as the mean ± SEM of three independent experiments (*lower panel*). β‐actin served as a loading control for whole‐cell lysate and cytosolic component. Cox4 served as a loading control for mitochondrial component. Figures presented are the representative figures of at least three independent experiments. Data were expressed as the mean ± SEM of three independent experiments. ns, non‐significant; ****P < 0.0001.

We further tested whether Dnm1l is required for cardiomyocyte mitochondrial fission by knocking down the endogenous Dnm1l expression using Dnm1l‐siRNA. As shown in Figure 6A, Dnm1l‐siRNA could significantly reduce the expression level of Dnm1l as analysed by immunoblot. Upon treatment with high concentration (2 μmol/l) of DOX, very few percentage of cells in knockdown group showed mitochondrial fission (Fig. 6B) relative to that of scrambled control as observed by mitochondrial staining. At the same time, we observed a reduction in DOX‐induced apoptosis in Dnm1l‐siRNA groups compared to its scrambled and negative control (Fig. S2A).

Others’ and our previous studies have shown that Dnm1l is translocated to mitochondria to initiate the mitochondrial membrane fragmentation upon exposure to cell stressors 23, 27, 28, 34. To understand the role of Dnm1l in the regulation of DOX‐induced apoptosis in HL‐1 cardiac myocytes, we performed subcellular fraction and compared the expression levels of Dnm1l between cytosolic and mitochondrial compartment before and after DOX exposure. Dnm1l was up‐regulated upon DOX exposure in whole‐cell lysate (Fig. 6C and D *upper panel*). Upon subcellular fraction analysis, we observed that Dnm1l was translocated from cytosol to mitochondria upon DOX treatment (Fig. 6C and D *middle and lower panels*). To understand whether Mtfp1 can influence Dnm1l translocation upon DOX exposure, we tested whether Mtfp1 knockdown can affect the Dnm1l recruitment in mitochondria. Our data showed that knockdown of Mtfp1 prevents the Dnm1l translocation into mitochondria even under high‐dose (2 μmol/l) DOX exposure (Fig. 6E and F). In addition, we found that Mtfp1 associated with Dnm1l upon DOX exposure and this association could be enhanced by Mtfp1 overexpression (Fig. S2B). Collectively, these data indicate that Mtfp1 is involved in the regulation of DOX‐induced Dnm1l accumulation in mitochondria.

## Discussion

The heart is a highly energy consuming organ and its function relies on its ATP‐generating systems, which is essentially governed by mitochondria 14, 15. Recently, it has been identified that mitochondrial morphology determines mitochondrial function as well as cell fate 35, 36. Mitochondria are highly dynamic structures that fuse and divide continuously to adjust the shape and distribution of the mitochondrial network depending on energy demand 36, 37, 38, 39. Excessive mitochondrial fission is involved in the initiation of apoptosis, while mitochondrial fusion can inhibit apoptosis 40, 41. Upon DOX exposure, mitochondria in HL‐1 cardiac myocytes transformed into punctate fragments and apoptosis initiation occurred 11, 13, 19. Hence, preventing mitochondrial fission could be a promising strategy in saving cardiomyocyte loss due to DOX‐induced cardiotoxicity 42. Here, we report the pro‐mitochondrial and pro‐apoptotic role of Mtfp1 in DOX‐induced cardiac toxicity, and knockdown of Mtfp1 can minimize the cardiomyocyte loss due to doxorubicin‐induced cardiotoxicity by preventing dyanmic 1‐like‐mediated mitochondrial fission.

Mtfp1 was first identified as downstream effector of PI‐3 kinase signalling. It comprises three ∝‐helical transmembrane domains and was found to localize in the mitochondrial inner membrane. The C‐terminus was shown to be essential for maintaining mitochondrial integrity and particularly predicted to participate in the regulation of mitochondrial fragmentation process in various cancer cells 24, 25. Mtfp1 is highly expressed in organs enriched with mitochondria such as skeletal and cardiac muscles 25, 43, 44; however, little is known about Mtfp1's role in cardiomyocytes’ mitochondrial dynamics. Additionally, the influence of Mtfp1 modulation on the mitochondrial fission event among different cell lines is controversial. In the human keratinocytes (HeCaT) cell line, Mtfp1 was reported to be essential for maintaining mitochondrial network and loss of Mtfp1's function was associated with extensive mitochondrial fission 25. In contrast, in the human cervical cancer cell lines, Mtfp1 is responsible for mitochondrial fission, and knockdown of Mtfp1 results in mitochondrial fusion 24. In our current study using HL‐1 cardiac myocytes, we found that Mtfp1 was up‐regulated upon DOX treatment and was associated with mitochondrial fission. Upon Mtfp1 knockdown, there was a significant inhibition of mitochondrial fission even when treated with a high dose of DOX. On the other hand, overexpression of Mtfp1 could induce a significant percentage of mitochondrial fission with suboptimal dose of DOX. Collectively, this study further confirmed the pro‐mitochondrial fission role of Mtfp1 in DOX cardiotoxicity.

Although the regulatory potential of Mtfp1 on mitochondrial dynamics has been documented, so far, only one study has investigated its association with apoptosis. The only study that we are aware of has proposed that reduction of Mtfp1 in HeCaT cells resulted in increased response to apoptotic stimuli, including UV and TNF‐α treatment 25. In contrast, our data revealed that Mtfp1 knockdown led to a resistance to DOX‐induced apoptosis in HL‐1 cells even when treated with a high dose of DOX. Together, these findings suggest a pro‐apoptotic role for Mtfp1 in DOX cardiotoxicity, which can be minimized upon Mtfp1 knockdown. In addition, these data highlight that protein regulating mitochondrial dynamics could function differentially between normal and cancer cells.

Dnm1l, which is also known as dynamin‐1‐like (Drp1), is a GTPase and is responsible for outer mitochondrial membrane scission. The Dnm1l‐mediated fission induces apoptosis by releasing cytochrome‐c (Cyt‐c) into cytosol 45. Multiple mitochondrial fission‐mediated proteins were found to influence Drp1 accumulation in the mitochondria upon cell stress. For instance, ARC prevents mitochondrial fission by associating with Dnm1l and preventing its translocation to mitochondria upon low‐dose DOX exposure in gastric cancer cells 46, or hFis1 promotes mitochondrial fission by enhancing Dnm1l accumulation in response to stress stimuli in mammalian cancer cells 47. On exploring the Mtfp1's target proteins with STRING program, our data showed that Dnm1l acquires the highest confidence interaction score among mitochondrial dynamic‐related proteins. Our loss of function study using Dnm1l‐siRNA showed that cardiomyocytes could not undergo efficient mitochondrial fission upon high‐dose DOX exposure, indicating that Dnm1l is essential for mitochondrial fission. In addition, we found that knocking down of Mtfp1 interferes with the Dnm1l accumulation in mitochondria, suggesting that Mtfp1 may associate with Dnm1l in the mitochondrial membrane and mediate the signal required for DOX‐induced mitochondrial fission and subsequent apoptosis.

Taken together, this study provides novel evidence that knocking down of Mtfp1 can minimize the cardiomyocyte loss in DOX‐induced cardiotoxicity, and thus, regulating its expression could be a novel therapeutic approach to treat chemotherapy‐induced cardiotoxicity. However, further *in vitro* studies in primary cardiomyocytes and *in vivo* experiments in cardiac models are required to identify the cardiac phenotype of Mtfp1.

## Conflict of interest

The authors declare no potential conflict of interests.

## Authors’ Contributions

Designed the experiments: L.H.H.A and P.L. Performed the experiments: L.H.H.A and R.L. Analyzed the data: L.H.H.A and P.L. Wrote the paper: L.H.H.A. Discussed, proof read and edited the paper: L.H.H.A, P.L. and B.S.P.

## Supporting information


**Figure S1**. Doxorubicin upregulates mitochondrial fission process 1 (Mtfp1) expression in whole cell lysate.
**Figure S2**. Dnm1l and Mtfp1 are involved in doxorubicin‐induced apoptosis.
