# Supplementary material for: Knockdown of Mtfp1 can minimize doxorubicin cardiotoxicity by inhibiting Dnm1l‐mediated mitochondrial fission
Source: J Cell Mol Med. 2017 Jun 23;21(12):3394–404. doi: 10.1111/jcmm.13250 (PMC5706585; doi:10.1111/jcmm.13250)
Supplement: Supplementary file 1 — Figure S1. Doxorubicin upregulates mitochondrial fission process 1 (Mtfp1) expression in whole cell lysate. Figure S2. Dnm1l and Mtfp1 are involved in doxorubicin‐induced apoptosis. [file JCMM-21-3394-s001.pdf]

Knockdown of Mtfp1 can minimize doxorubicin cardiotoxicity by inhibiting Dnm1l-mediated mitochondrial fission

Lynn H.H. Aung[1], Ruibei Li[2], Bellur S. Prabhakar[1], Peifeng Li[1†]

[1]*Department of Microbiology and Immunology, College of Medicine, University of Illinois at Chicago, Chicago, IL, USA*

[2]*School of Professional Studies, Northwestern University, Chicago, IL, USA*

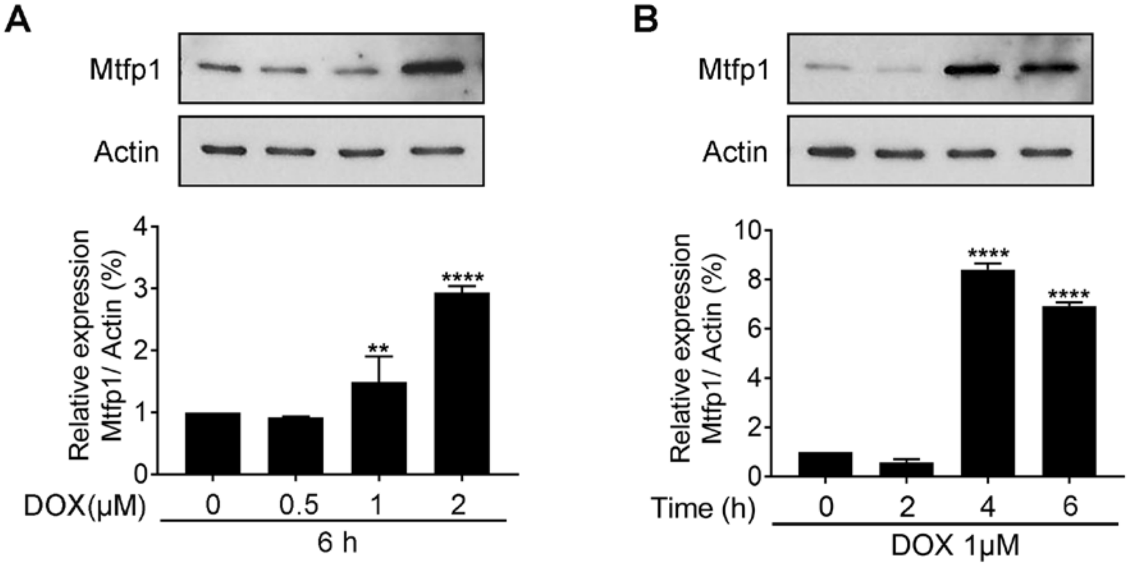

Figure S1. Doxorubicin upregulates mitochondrial fission process 1 (Mtfp1) expression in whole cell lysate. A and B. Analysis of Mtfp1 expression by immunoblot. HL-1 cells were stimulated with the indicated doses of DOX and harvested at 6hr (A), and cells were stimulated with 1μmol/L DOX and then harvested at the indicated time (B) for immunoblotting (*upper panel*). Actin served as a loading control. Figures presented are representative of at least three independent experiments. The densitometry data are presented as the mean ± SEM of three independent experiments (A and B *lower panel*). $^{**}P<0.01$, $^{****}P<0.0001$ *vs* non-treatment.

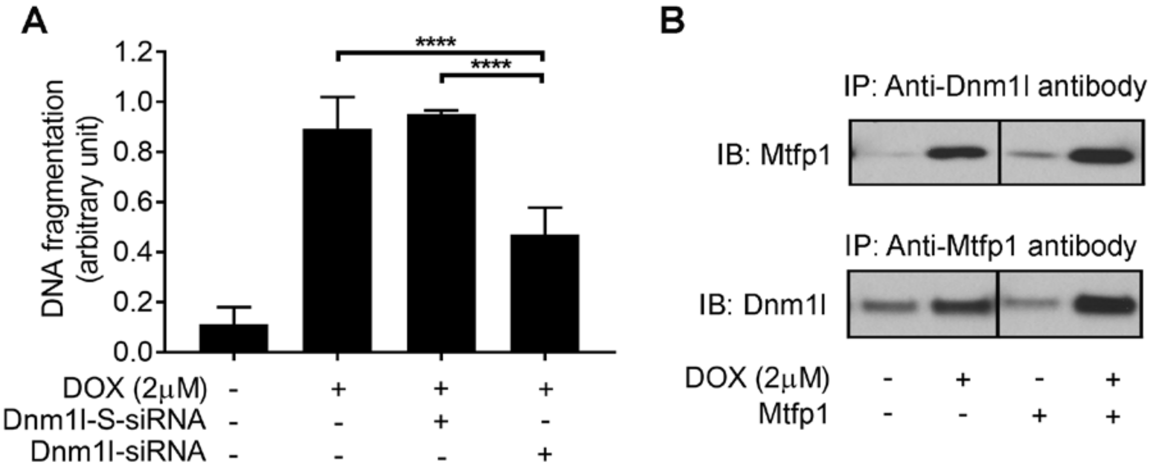

Figure S2. Dnm1l and Mtfp1 are involved in doxorubicin-induced apoptosis. A. Knockdown of Dnm1l prevents doxorubicin-induced apoptosis**.** HL-1 cells were transfected with 30nM Dnm1l siRNA. After 48hr, cells were treated with doxorubicin (DOX) for 9hr and apoptosis related DNA fragmentations were analyzed using the cell death detection ELISA. Data were expressed as the mean ± SEM of three independent experiments. ****P<0.0001. B. Mtfp1 associates with Dnm1l upon DOX exposure. Cells were infected with Lentiviral-Mtfp1 and after 24hr, treated with 1μmol/L DOX. The cells were harvested after 6hr of DOX treatment and the association between Mtfp1 and Dnm1l was analyzed by immunoprecipitation (IP) followed by immunoblot (IB). Figures presented are representative of at least three independent experiments.
